# Amide/Amino-Based Functional Additives for Lubricants: Structure, Antimicrobial Activity and Wear Resistance

**DOI:** 10.3390/molecules29010122

**Published:** 2023-12-24

**Authors:** Jolanta Drabik, Kamil Korasiak, Justyna Chrobak, Julia Woch, Natalia Brzeźniak, Wioletta Barszcz, Rafał Kozdrach, Jolanta Iłowska

**Affiliations:** 1 Łukasiewicz Research Network–Institute for Sustainable Technologies, 26-600 Radom, Poland; jolanta.drabik@itee.lukasiewicz.gov.pl (J.D.); wioletta.barszcz@itee.lukasiewicz.gov.pl (W.B.); rafal.kozdrach@itee.lukasiewicz.gov.pl (R.K.); 2 Łukasiewicz Research Network–Institute of Heavy Organic Synthesis “Blachownia”, 47-225 Kędzierzyn-Koźle, Poland; kamil.korasiak@icso.lukasiewicz.gov.pl (K.K.); julia.woch@icso.lukasiewicz.gov.pl (J.W.); natalia.brzezniak@icso.lukasiewicz.gov.pl (N.B.); jolanta.ilowska@icso.lukasiewicz.gov.pl (J.I.)

**Keywords:** lubricants, antibacterial properties, anti-wear properties, oxidation stability

## Abstract

The lubricating properties of the lubricants were tested under boundary friction conditions; it was found that the surface-active additives had reduced the wear mark and thus the value of the Goz/40 parameter (limiting load of wear). The introduction of a surfactant containing amide compounds into the oils and greases was highly effective in slowing down the oxidation process. Lubricants containing mono–15 ([3-(*N*,*N*,*N*-dimethylbuthylamine)propyl]hexadecanamide chloride) and 15–4–15 (tetramethylene-bis [3-(*N*,*N*,*N*-dimethylamine)propyl]hexadecanamide) additives were characterised by higher oxidation stability compared to the unmodified lubricants. Both of the analysed substances showed bactericidal properties against *Staphylococcus aureus* and *Salmonella enteritica*. Tests of antibacterial activity in the lubricants with the addition of mono–15 and 15–4–15 confirmed that these lubricants can be considered bactericidal against Gram-positive and Gram-negative bacteria.

## 1. Introduction

The characteristics of lubricating greases and oils are determined by their formulation and production processes and are affected by, in particular, appropriately formulated fortifying agents [1,2,3,4]. The most common modifiers used to improve the characteristics of greases and oils contain oxidation inhibitors, friction and galling inhibitors, corrosive inhibitors, adhesives and rheology modifiers [1,5,6,7,8,9]. However, the practical behaviour of the grease and oils is dependent not just on the existence of the modifier, but also on the way in which the modifier is introduced to the grease and oil composition. The incorporation of modifiers in the lubricating greases and oils poses various technical problems due to the adsorption of the modifier particles on the thickening material, which can reduce the efficiency of this component and actually reduce the durability of the grease and oils [1,3,5,7,10,11].

Suitably chosen modifiers need to be added to grease and oils in amounts that will provide enhanced performance. Lubricating greases and oils blend easily with solid-lubricating modifiers, which decrease friction and improve the ability of the sliding combination to resist stress and scuffing [12,13,14,15,16,17]. In extreme operating regimes, such lubricant modifiers can improve the performance of the grease and oil by resisting the effects of chemical degradation and improving their ability to withstand exposure to extreme heat. The most popular of these modifiers are silica, molybdenum disulphide, and polytetrafluoroethylene [5,6,8,9,18,19,20,21,22]. The incorporation of additives into the composition of the grease greatly increases the lubricating characteristics, protection against scuffing and wear, and the physicochemical and rheology parameters on which grease and oil composition efficiency is directly dependent [12,13,14,15,16,17,20,21,22,23].

Modifying additives are substances in solid or liquid form that are introduced into grease or oil to improve their functional properties [1,2,3,4]. Typically, the additives are introduced into the structure of the lubricants in an amount of several percent [5,6,7,8,9,14,16]. This amount of modifier significantly improves the properties of the grease or oil compared to the base oil or grease (without the modifier).

Aliphatic hydrocarbons are used in triboactive additive performance tests. They affect the resistance of lubricants to seizure and wear processes and have a particular influence on additive changes during tribological processes [24,25]. The interactions that occur between the steel components of a friction junction when the base oil comes into contact with different additives were evaluated [26].The influence of lubricity additives added to paraffin oil on the tribological properties of the sialon-cast iron system was evaluated [27], and tribological transformations of the additive during the friction and wear process [28,29,30]. The effect the additive particles adsorbing on the thickener particles had on the tribological properties was evaluated [31], and the role of multifunctional additives in the friction process using bentonite-thickened grease [32]. The function of nanoparticles of inorganic fullerenes in the friction process [33] and the behaviour of nanoparticles of the additive in oil during the friction process [34] were investigated. The compatibility of the additive and thickener with respect to changes in the structure of the lubricant during tribological processes is very important [35]. Silica additives are of great tribological and rheological importance for organic greases under conditions of extreme tribological forces [36,37]. Polymeric additives are also important for friction and wear processes [38]. The use of ecological viscosity additives that are safe for the natural environment is of great importance for the development of modern science [39].

Classic lubricants contain components that fulfil high operational requirements, but most often do not meet the standards for an ecological lubricant. For this reason, the main task is to develop and test new, non-toxic and biodegradable lubricants based on chemical compounds, which would provide a high-quality product both in terms of operation and ecology. The biggest problems are additives which, when used in this type of lubricant, should not contain heavy metals and should be free of any carcinogenic compounds [7].

The increasingly wide spectrum applications of lubricants in the food industry require effective and efficient agents whose properties must be consistent with the requirements of the European Union in the field of safety and hygiene in food production. In terms of EU legislation, the priority in the field of lubricants intended for use in food machines is to develop them with the elimination or significant reduction of toxic components. These requirements completely or partially eliminate lubricants that have been used in the food industry for many years and which, although effective, did not meet the requirements for safe food production [9].

Most often, non-toxic substances that do not cause degradation of the natural environment are used for greases used in the food and agricultural industry [18,20,22]. Such additives are calcium borate [10], graphene [11], carbon nanotubes [12], polyvinylpyrrolidone [13], calcium salicylate [15], graphite [17], polytetrafluoroethylene [19], chitosan [21], montmorillonite and silica [23].

The awareness of environmental threats determines the need to develop lubricants based on alternative raw materials and petroleum substitutes. The above-mentioned factors have caused significant qualitative and quantitative changes in the demand for natural oils that can be successful substitutes for crude oil [1]. Moreover, replacing crude oil with other raw materials is needed due to the fact that petroleum oils and lubricants obtained based on them pose a high risk of contamination of soil and groundwater. In this situation, carrying out work on the development of new technologies for the production of lubricants based on vegetable oil bases and unconventional additives is of significant importance and is considered crucial.

**Figure 1:**
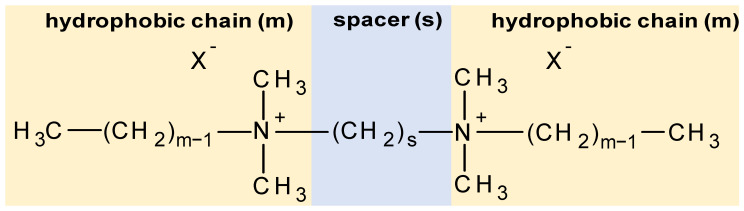
General structure of gemini QACs.

Quaternary ammonium compounds (QACs) are a group of chemicals widely used in many branches of industry, for instance as softeners [40], dyes [41], corrosion inhibitors [42], etc. One of the groups of QAC are gemini compounds, which are “double” ammonium salts containing twin quaternary structures bonded by a common alkyl chain known as a “spacer”. Regarding single-chain QACs, gemini QACs are more effective in reducing surface tension; they also have better wetting properties and often lower critical micellisation concentrations (CMCs) [43]. The general structure of gemini compounds (Figure 1) is often described in the simplified form of “m–s–m”, where “m” is the number of carbon atoms in the hydrophobic chain and “s” is the number of carbon atoms in the spacer.

QACs contain positively charged nitrogen atoms; therefore, they can easily adsorb onto negatively charged surfaces [44]. Thus, QACs are able to adsorb onto a microbe surface, enter the lipid layer of a cell membrane, and damage the cell. Antimicrobial activity depends on the adsorption capability of the QAC on a microorganism cell surface, its hydrophyllic–lipophyllic balance (HLB), and its alkyl chain length. As antibacterial agents, gemini QACs can be used in lower concentrations than conventional “single” QACs because gemini QACs generally have a lower CMC (critical micellisation concentration) than conventional surfactants, as well as better antibacterial activity [45].

The influence of the spacer and chain lengths on the gemini QACs’ antimicrobial properties was studied by Laatris, A. et al. [46], as shown in Table 1.

Kuperkar, K. et al. conducted a more comprehensive study including bacterial strains, as well as one fungal strain. The results of their study are shown in Table 2.

The results above show that gemini QACs with longer hydrophobic chains and shorter spacers exhibited greater antimicrobial activity.

**Figure 2:**
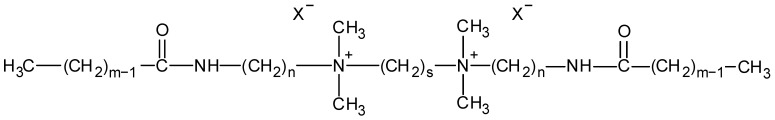
General structure of gemini amide/QACs.

Ghumare, A.K. et al. investigated gemini amide/QACs (Figure 2) in comparison with monomeric amide/QACs. The results of their study are shown in Table 3.

The results above show that gemini QACs exhibited greater antimicrobial activity against *P. aeruginosa* and *B. subtilis* than their monomeric QACs counterparts with the same alkyl chains.

In addition to their antimicrobial properties, QACs are a promising group of corrosion inhibitors because of their ability to absorb onto a metal surface and create a protective layer.

The present research proposes a method for the synthesis of QACs in the form of “double” ammonium salts and offers a comparison of their antibacterial and tribological properties with those of the monomeric analogue. It was assumed that these compounds, when added to the vegetable grease, would improve its functional properties and increase its resistance to the development of Gram-negative and Gram-positive bacteria. The synthesised additives were marked as follows: 15–4–15, which is amidoammonium chloride (gemini), and mono–15 [3-(*N*,*N*,*N*-dimethylbutylamino)-propyl] chloride hexadecanamide. The antibacterial properties of surface-active additives in the form of branched amines were tested against two strains of bacteria: *Staphylococcus aureus* (*S. aureus*) and *Salmonella enteritica* (*S. enteritica*). Based on the minimal bactericidal concentrations of the analysed compounds, antibacterial tests of the grease were carried out. These studies were intended to verify whether the prepared QACs increase the grease’s resistance to the formation of bacterial biofilms of *S. aureus* and *S. enteritica.* According to the literature, these types of surfactants can exhibit antibacterial and cytotoxic properties [49,50]. In order for surfactants to be effective against bacteria, they must be able to adsorb to the cytoplasmic membrane [49]. The action mechanism is based on the interaction between a positively charged group of surfactants and a negatively charged membrane of bacterial cells. This interaction causes selective permeability disorders in the cell membrane, eventually leading to cell death [51,52]. The microbiological tests were carried out for the developed functional vegetable grease (Grease A) containing the appropriate weight percentage of the tested additives (Grease A + mono–15, Grease A + 15–4–15).

In addition to microbiological tests, tribological tests were carried out to determine the anti-wear properties, with the aim of identifying the effect of the additives on the lubricating properties of the lubricants. The tests were carried out on the PetroOxy apparatus, which made it possible to determine the effect of the additives on the modification of the oxidative stability of the lubricants under consideration.

## 2. Results

**Figure 3:**
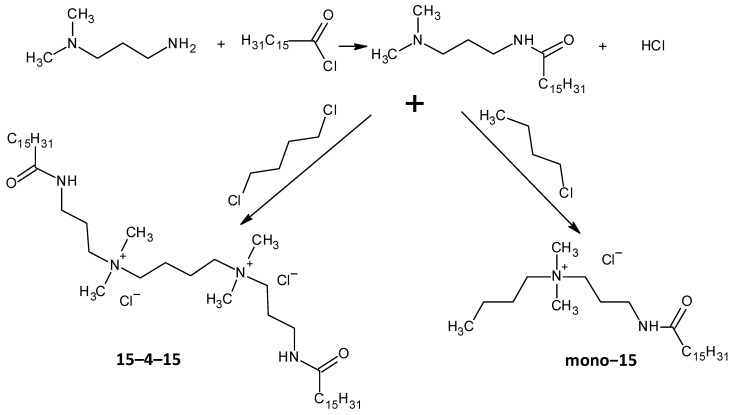
Scheme of the synthesis of the investigated QACs.

Two QACs were synthesised: [3-(*N*,*N*,*N*-dimethylbuthylamine)propyl]hexadecanamide chloride (further marked as mono–15) and tetramethylene-bis [3-(*N*,*N*,*N*-dimethylamine)propyl]hexadecanamide chloride (further marked as 15–4–15). All synthesis routes are shown in Figure 3.

### 2.1. Analysis of Synthesised QACs

#### 2.1.1. [3-(*N*,*N*-dimethylamine)propyl]hexadecanamide

**Figure 4:**
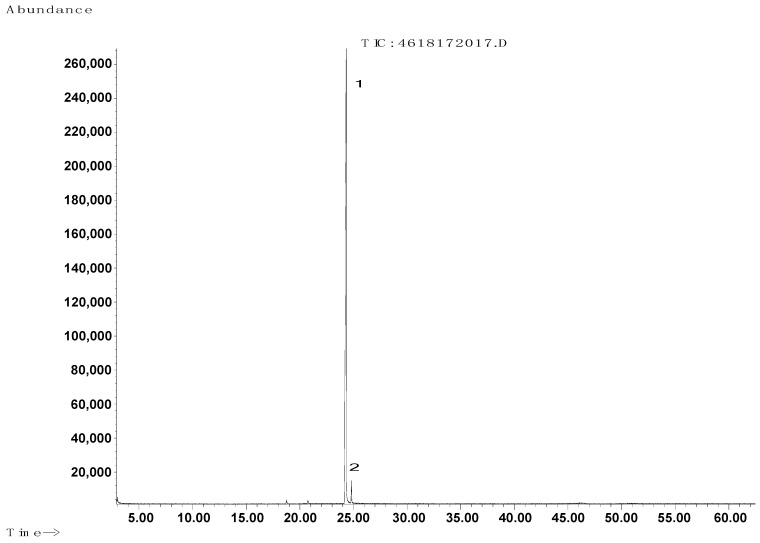
Chromatogram of obtained [3-(*N*,*N*-dimethylamine)propyl]hexadecanamide.

**Figure 5:**
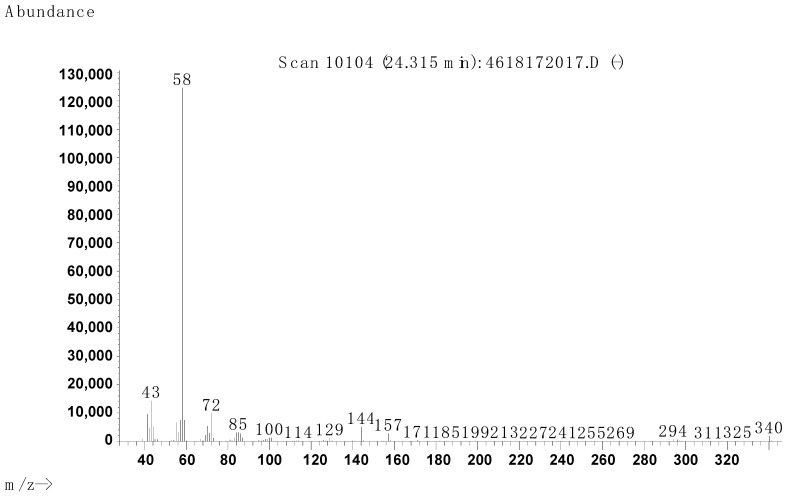
Mass spectrum of the main product (1) obtained from GC.

**Figure 6:**
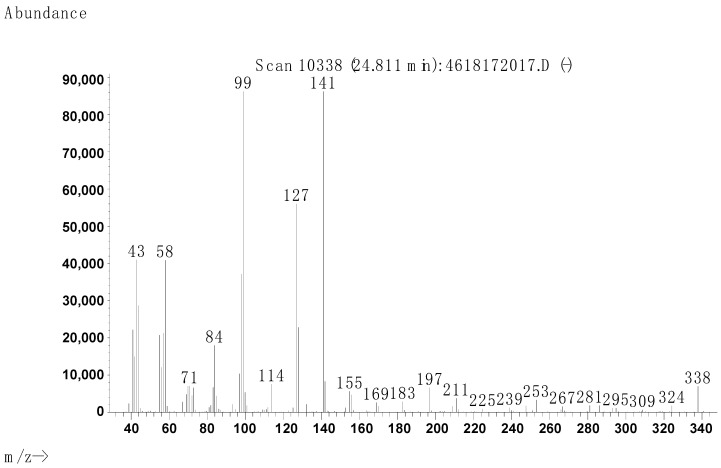
Mass spectrum of the secondary product (2) obtained from GC.

The synthesis procedure is described in Section 4.2.1. The purity was determined via GC/MS with a result of 97.6% (Figure 4, Figure 5 and Figure 6, Table 4).

#### 2.1.2. 15–4–15 Gemini QAC

**Figure 7:**
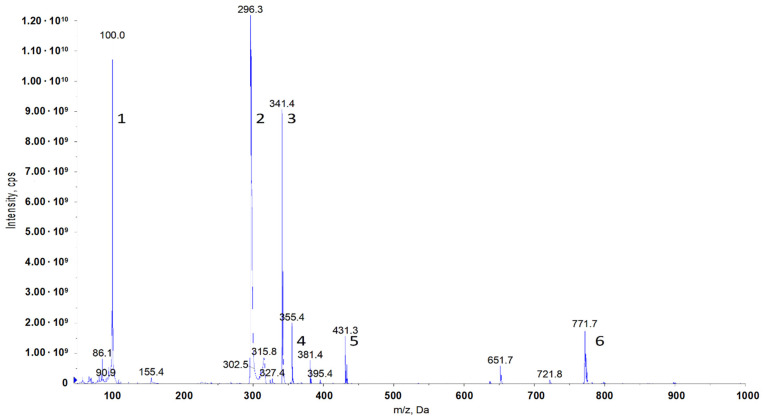
Mass spectrum of the obtained 15–4–15 QAC.

The synthesis procedure is described in Section 4.2.2. The structure was confirmed via ESI/MS (Figure 7, Table 5).

#### 2.1.3. Synthesis of mono–15 QAC

**Figure 8:**
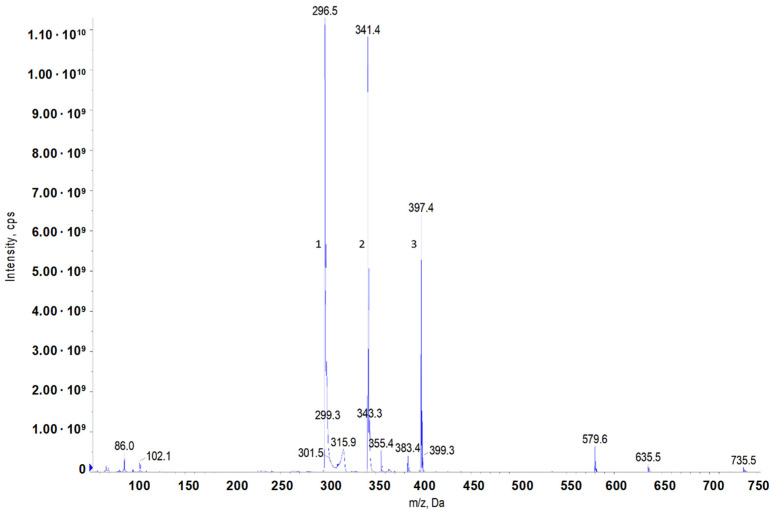
Mass spectrum of the obtained mono–15 QAC.

The synthesis procedure is described in Section 4.2.3. The structure was confirmed via ESI/MS (Figure 8, Table 6).

### 2.2. Antibacterial Activity

#### 2.2.1. The Results of the Determination of Antibacterial Properties of mono–15 and 15–4–15 Additives

**Figure 9:**
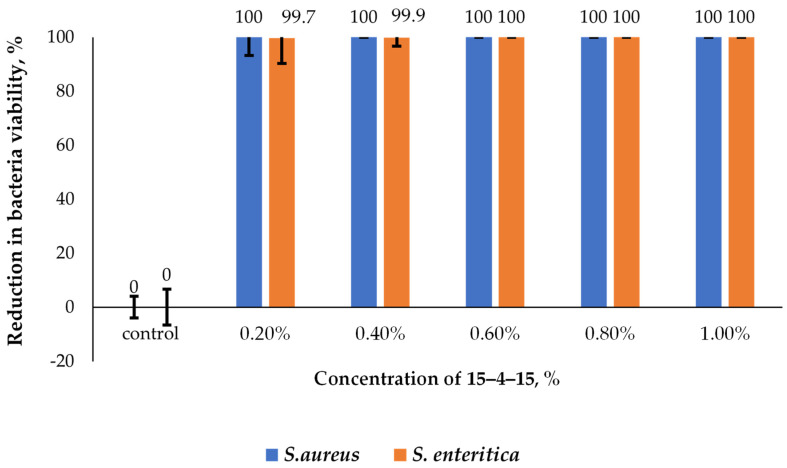
Comparison of the effect of the 15–4–15 (gemini) concentration on the growth of *S. aureus* and *S. enteritidis*.

Two types of surface-active additives were analysed, 15–4–15 and mono–15, which were used to prepare the solutions of different concentrations in accordance with the described methodology for the assessment of surface-active additives. The lowest bactericidal concentrations against strains of *S. aureus* and *S. enteritica* were determined. The minimum bactericidal concentration is the concentration of the solution that inhibits the growth of bacteria at the level of 99.9%. Figure 9 shows the results obtained for different concentrations of 15–4–15.

In the case of bactericidal activity against *S. aureus*, in the concentration range of 0.20–1.0%, no live cells of this microorganism were detected. The bactericidal effect was also observed against *S. enteritica* bacteria in the concentration range of 0.20–1.0%, and an increase in the concentration of the additive to 0.60% led to the inhibition of the growth of this microorganism to >99.9%.

**Figure 10:**
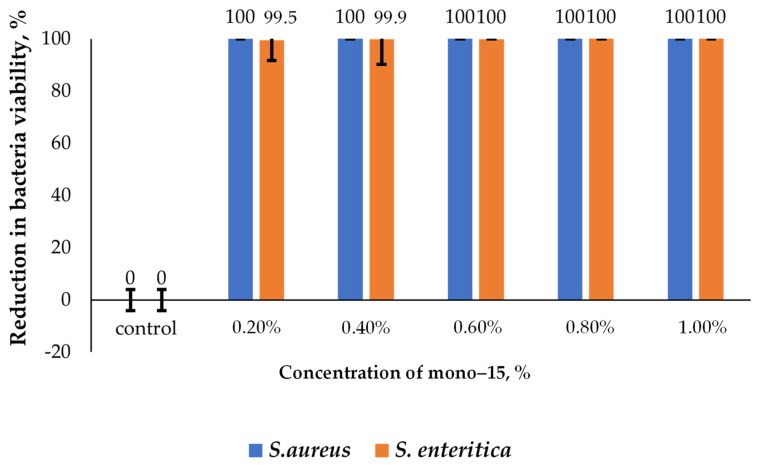
Comparison of the effect of the mono–15 concentration on the growth of *S. aureus* and *S. enteritidis*.

In the case of mono–15, the obtained results indicate a bactericidal effect that is comparable to the activity of 15–4–15 against the analysed strains; see Figure 10. The minimum bactericidal concentration against *S. aureus* was achieved at a concentration of 0.20%, and at a concentration of 0.4% against *S. enteritica*. The obtained results indicate that both of the analysed surfactants have an effective bactericidal effect against Gram-positive and Gram-negative bacteria.

In conclusion, in the experiments conducted to assess the bactericidal effect of the surface-active additives against Gram-positive *S. aureus* and Gram-negative *S. enteritica* bacteria, a bactericidal effect was confirmed for both of the additives analysed.

#### 2.2.2. The Results of the Determined Antibacterial Properties of Grease with mono–15 and 15–4–15 Additives

**Figure 11:**
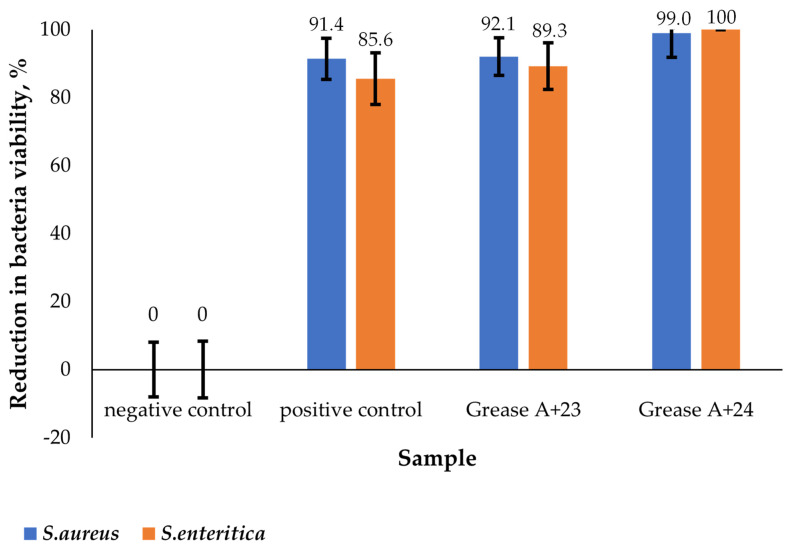
Comparison of the bactericidal properties of greases with a 0.2% addition of surfactants, Grease A + (mono–15), and Grease A + (15–4–15), against *S. aureus* and *S. enteritica*.

Then, according to the described procedure, the antibacterial properties of vegetable Grease A modified with surface-active additives (Grease A + mono–15 and Grease A + 15–4–15) were tested against *S. aureus* and *S. enteritica*, as shown in Figure 11.

The analysis of the obtained results indicates that the addition of the additives increased the antibacterial effectiveness. The growth of the tested bacteria was inhibited by the grease without additives. The grease without additives (positive control) showed 85.63% and 91.43% effectiveness in reducing the growth of *S. enteritica* and *S. aureus*, respectively. The results obtained for the grease without the addition of mono–15 or 15–15 allow us to conclude that it has bacteriostatic properties. The antimicrobial efficacy against both strains was confirmed for the Grease + 15–4–15 sample. The use of this additive made it possible to achieve a bactericidal effect against *S. aureus* at the level of 99.0109% and against *S. enteritica* at the level of 99.9999%. For the Grease A + mono–15 sample, the effectiveness of the inhibition of bacterial growth was similar to that detected for the grease sample without the additive (positive control). The presence of a Grease A + mono–15 sample in the environment made it possible to reduce the growth of *S. aureus* to the level of 92.09% and *S. enteritica* to the level of 89.27%.

To sum up, the surface-active amide/amine additives 15–4–15 and mono–15 have a bactericidal effect against Gram-positive and Gram-negative bacteria. The lowest bactericidal concentration needed to obtain a bactericidal effect of additive 15–4–15 for *S. aureus* was 0.20%, and for *S. enteritica* it was 0.40%. The lowest bactericidal concentration of additive mono–15 for *S. aureus* was 0.20%, and for *S. enteritica* it was 0.40%.

This research on the bactericidal properties of greases with surface-active additives (15–4–15 and mono–15) against *S. aureus* and *S. enteritica* bacteria allows us to conclude that the modification of the grease with the additives contributed to the more effective inhibition of the growth of these microorganisms in relation to the basic grease. At the same time, it is worth noting that the basic grease already had a strong bacteriostatic effect (growth reduction by approx. 90%). However, these results indicated that by introducing additives in the form of QACs, we can increase the usability of the final product.

### 2.3. The Results of the Determination of Additives’ Effectiveness in the Environment of Oil and Vegetable Grease

Lubricating Grease A contains Oil A and inorganic thickener. Grease A parameters: dynamic viscosity—4.46 [Pa·s] at 40 [°C]. Oil A parameters: kinematic viscosity at 40 [°C]—47.64 [mm^2^/s], density at 40 [°C]—0.902 [g/cm^3^].

The dynamic viscosity test was carried out using an MCR 101 rotational rheometer from Anton Paar according to our own methodology, while the kinematic viscosity test was carried out using an automatic Ubbelhode HVU 481 viscometer from Herzog according to the methodology described in the PN-EN ISO 3104:2021-03 standard.

The developed additives were intended to enhance the bactericidal effect of the lubricants, which was confirmed by microbiological tests. Then, oil and grease samples containing those additives were tested to assess their effect on lubricating and anticorrosive properties and oxidation stability (Table 7).

The lubricating properties were assessed in relation to the value of the limit wear load G_oz_/40 and the value of the average diameter of the wear mark d after an hour-long test under a load of 392.4 N. Tribological tests were carried out at temperature of 20 °C, and the concentration of both additives in the lubricants was 0.2%. For each sample, tribological tests on four-ball apparatus (T-02, produced by the Łukasiewicz Research Network—Institute for Sustainable Technologies in Radom) were carried out in three repetitions.

During the measurement, the tribosystem was not heated, but during the test the temperature of tribosystem increased to an average temperature of 33.8 °C for oils and 29.9 °C for greases.

In the experiment pure base oil (Oil A) and grease based on base Oil A and an inorganic thickener were used. Oil A and Grease A did not contain additives.

Each change in the composition of the lubricant requires a series of tests to determine the change in its lubricating properties and oxidation and anti-corrosion resistance. Additives used reduced the diameter of the friction scars of the steel elements, which proves the effective action of both additives. The subject of the research was to check the possibility of using developed additives as unconventional bactericidal additives in vegetable lubricants.

The analysed additives had antibacterial properties, and tribological tests confirmed that the scar diameter of the evaluated lubricating compositions indicates a low value of wear, which is reflected in the limiting load of wear value G_oz_/40. Additives used were supposed to have a bactericidal function, but during tests it was found that they provide anti-wear and anti-corrosion protection. Improving the anti-wear properties of oils and greases is a positive effect of the research carried out. The surface was not evaluated using the SEM-EDS technique, because the additives used were not lubricating additives, and there was no discussion of lubrication mechanisms, which will be the subject of further research.

**Figure 12:**
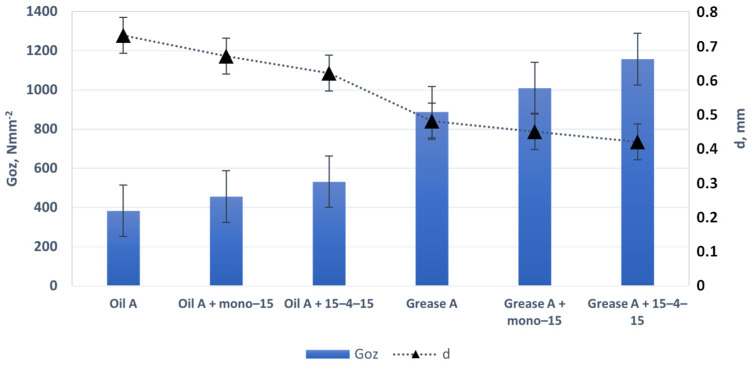
The limit wear load (G_oz_) and diameter of the wear mark (d) of lubricants containing mono–15 and 15–4–15 additives.

The wear load limit value (G_oz_) characterises the anti-wear properties of the grease under boundary lubrication conditions. It was observed that, under boundary lubrication conditions, the most favourable anti-wear properties were found in the grease containing the additive 15–4–15 (Figure 12).

The copper plate after the test on corrosion was compared to the corrosion standard scale and the level of corrosion was determined on a scale of 1–4 and subcategories from **a** to **e** (according to the PN-EN ISO 2160:2004). The obtained results of corrosion tests on copper plates confirmed that Oil A and Grease A did not cause corrosion on copper. The modification of grease and oil with the additives used did not cause any changes in corrosion.

**Figure 13:**
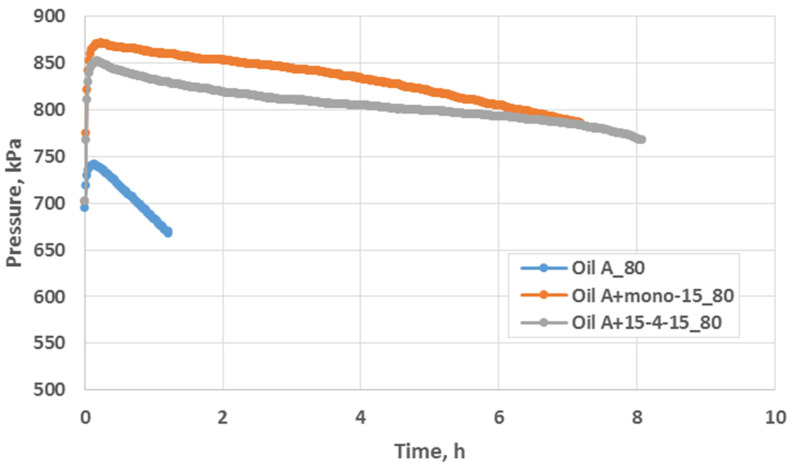
The influence of the additives (mono–15, 15–4–15) on the oxidation stability of oils according to PetroOxy at 80 °C.

The oxidation stability was determined on the basis of the accelerated oxidation test using the PetroOxy method. The oxidation induction time (Figure 13) was determined based on the obtained graphs of the pressure drop as a function of the oxidation time, proving the resistance to the oxidation process of the tested lubricants.

**Figure 14:**
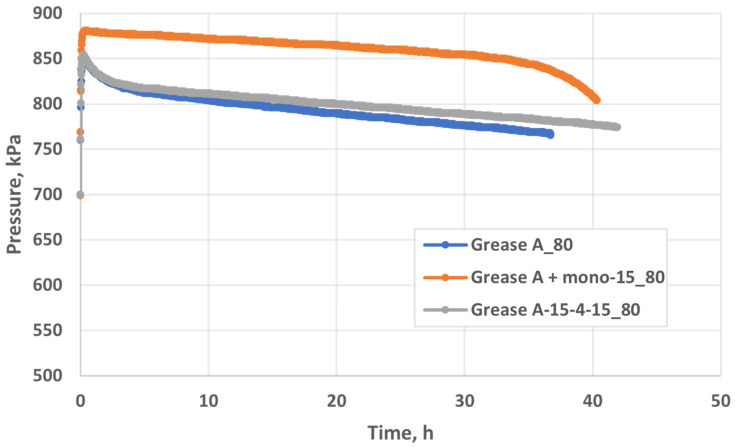
The influence of the additives (mono–15, 15–4–15) on the oxidation stability of Grease A according to PetroOxy at 80 °C.

As an example, a course of oxidation processes at a temperature of 80 °C was carried out using the PetroOxy apparatus for base oil and for oils modified with developed additives, on the basis of which the oxidation induction time was determined. The oxidation tests were also carried out for lubricants (Figure 14), and Table 7 shows the final result of the oxidation process (the oxidation induction time of lubricating compositions at a temperature of 80 °C).

## 3. Discussion

QACs have been used as antimicrobial agents in products or for infection control for over 60 years. In the food industry, they are widely used to limit the growth of bac-teria such as *Escherichia coli* or *Listeria monocytogenes* [53]. The antimicrobial properties of quaternary amine compounds mainly depend on the length of the n-alkyl chain, as has been confirmed in many studies [46,54,55].

The mechanism of antimicrobial action of QACs is most likely related to the interaction of the lipophilic chain and cations on the negatively charged cell membrane of microorganisms [56,57,58,59]. The bactericidal effect of QACs takes place over several stages. The adsorption of cations to the membrane is one of the first and most important steps, and it is based on electrostatic interactions and binding to the membrane. As a consequence, the cell membrane is destabilised, which causes its breakdown and the excessive leakage of intracellular substances through the damaged spot. Cell death is an inevitable result of this process. Compared to antibiotics, QACs have a more effective bactericidal effect. The mechanism of action of antibiotics is to bind to a specific substance contained in the bacterial cell membrane. In the case of QACs, due to their ability to incorporate a lipophilic chain into the cell membrane, they cause its breakdown and, consequently, the death of the cell [60].

The bactericidal effect of QACs is stronger against Gram-positive bacteria than Gram-negative ones [61]. It can be assumed that this is due to differences in the structure of the cell membrane: in Gram-negative bacteria, murein is surrounded by an additional outer membrane composed of proteins, phospholipids, and lipopolysaccharide (LPS), which determines their sensitivity to chemicals and antibiotics [62]. We obtained two QAC compounds, mono–15 and 15–4–15 which were confirmed by the MS spectra. The tests carried out using grease with the addition of synthesised mono–15 and 15–4–15 QACs against *S. aureus* and *S. enteritica* bacteria showed that slightly better bactericidal properties are obtained against Gram-positive bacteria than Gram-negative ones. However, it should be noted that 15–4–15 shows higher activity than mono–15 against both bacteria (Figure 11). Devinsky et al. showed that the bactericidal effect is related to the length of the alkyl chain. Initially, as the length of the chain is extended up to *n* = 10–14, the concentration of QACs required to effectively limit the growth of microorganisms may be minimal. However, further increasing the chain length reduces the solubility of the compounds and consequently reduces their bactericidal activity [63,64]. The bactericidal effect against Gram-positive bacteria is greater for compounds with *n* = 12–14 chains and against Gram-negative bacteria for *n* = 14–16 chains. The results obtained for the QAC compounds analysed in this work show that, for both types of bacteria, the ability to proliferate cells was reduced by over 99% for the lowest concentration (0.2%, see Figure 9 and Figure 10). Kowalczyk et al. analysed the antimicrobial properties of QACs containing a double ammonium cation and a double alkyl chain. It has been shown that, to effectively limit the growth of fungi and bacteria, a lower concentration of this type of agent can be used in comparison to the standard compounds with one ammonium cation and a single alkyl chain [65]. Koziróg and Brycki showed that hexamethylene-1,6-bis-(*N*,*N*-dimethyl-*N*-dodecylammonium bromide) exhibits greater antibacterial activity than its monomeric analogue, even at concentrations 17–70 times lower [66]. The bactericidal effect of surfactants depends on the hydrophobicity of the n-alkyl chain. Additionally, considering the source of the alkyl substituent, it can be concluded that antibacterial activity is different for different compounds [67,68,69]. The study showed that the addition of a QAC with a double ammonium cation and a double alkyl chain exhibits more pronounced antibacterial activity against *S. aureus* and *S. enteritica* than the mono QAC. Thus, the original assumptions were confirmed, and it can be stated that the use of a lubricant with the addition of synthesised QACs in the food industry can significantly improve the microbiological safety of the production area.

When assessing the lubricating properties of lubricants under boundary friction conditions, it was found that surface-active additives reduce the wear mark and thus the value of the wear limit load. It was found that, in both the oil and grease environments, the additives exhibit anti-wear properties. The experiments conducted indicate that the introduction of a surface-active additive containing amide compounds to oil and grease has a pronounced effect in terms of slowing down the oxidation process. Greases containing additives (mono–15 and 15–4–15) are characterised by higher oxidation stability compared to unmodified oil and grease. It was found that the additives introduced into the oil and lubricant significantly affect the extension of the oxidation induction time, which confirms the increase in the oxidative stability of the assessed agents (Figure 13). Particularly in the case of oil, a significant increase in the oxidation induction time (approx. six times) was observed in relation to unstabilised oil. This proves that the developed additives act as oxidation inhibitors in a vegetable oil environment.

## 4. Materials and Methods

### 4.1. Analytical Methods

#### 4.1.1. Gas Chromatography

We used a Hewlett-Packard HP 6890 Series GC System gas chromatograph equipped with an MSD 5973 Network mass spectrometry detector and a Chemstation computer.

Chromatographic separation was performed using a capillary chromatographic column made of fused silica; it was 25 m long and with an internal diameter of 0.2 mm and filled with a stationary phase of the HP5MS type with a film thickness of 0.2 µm. During the analysis, the column was heated in the temperature range of 70–250 °C at a linearly programmed rate of 7 °C/min. The temperature of the split/splitless dispenser was 300 °C, and the flow of helium, which was the carrier gas, was set to 0.5 mL/min. The split ratio of the carrier gas and the analysed sample in the dispenser was set to 100:1. The tested sample was dosed onto the chromatographic column in the form of a chloroform solution in an amount of 0.1 µL using a Hamilton microsyringe.

Mass spectrometry was used to confirm the structure of the compounds. After separating the components in the chromatographic column, mass spectra were obtained under the following conditions: GC/MS transfer line temperature = 280 °C; ion source temperature = 180 °C; ionisation energy of the electron beam = 70 eV; ion acceleration voltage = 1500 V; m/z range: 14–550 Da.

#### 4.1.2. Mass Spectrometry

Samples were analysed with the use of the AB-Sciex triple quadrupole mass spectrometer, model Q-TRAP 4000. Spectra were obtained in the positive ion mode. Electrospray (ESI) was used as the ionisation method. The test sample was dissolved in methanol using a 100-fold dilution.

#### 4.1.3. Antibacterial Properties

Microbiological tests were performed against Gram-positive *Staphylococcus aureus* (*S. aureus*; ATCC 25923) and Gram-negative *Salmonella enteritica* (*S. enteritica;* ATCC 13314). The strains came from the American Type Culture Collection.

##### The Antibacterial Properties of Surfactant Additives

Sterile flasks containing 10 cm^3^ of Mueller–Hinton liquid (MHB, VWR) were inoculated with a single colony of the selected bacterial strains and incubated at 37 °C overnight with shaking at 240 rpm. The analysed additives were diluted in a water/alcohol solution (3:1 *v*/*v*), and then a series of solutions were prepared with concentrations of 0.20%, 0.40%, 0.60%, 0.80%, and 1.0%. The inoculum was prepared via the dilution of an overnight culture of the selected bacterial strains to the concentration of (1.5–3.0) · 10^8^ CFU/cm^3^ (CFU, colony forming unit) in the sterile MHB growth medium. The concentration of the inoculum was verified by spectrophotometric measurement (Hach, DR6000) at a wavelength of 600 nm. Then, 9 cm^3^ of the liquid MHB medium, 0.9 cm^3^ of a surfactant additive solution, and 0.1 cm^3^ of the inoculum were transferred to sterile flasks. The prepared samples were incubated at 37 °C overnight. The control sample was a sterile MHB medium containing 0.9 cm^3^ of sterile phosphate buffer and 0.1 cm^3^ of inoculum with a concentration of (1.5–3.0) × 10^8^ CFU/cm^3^. Afterwards, inoculations were prepared via the serial dilution method on the plates containing the growth medium TSA (tryptic soy agar, VWR). Plates were incubated at 37 °C for 24 h. After this time, the plates were analysed and it was determined whether superficial colony growth was visible on their surface, and the number of colony-forming units was counted. The analyses were performed three times, and then the arithmetic mean was calculated from the obtained concentrations. The results were given as a percentage of reduction compared to the control sample in accordance with the formula

(1) $$R=\frac{I_{w}\;-I_{p}}{I_{w}}\cdot 100\%$$

where *I_w_* is the concentration of the microorganism in the control sample [CFU/cm^3^], and *I_p_* is the concentration of the microorganism after contact with the tested sample [CFU/cm^3^].

The analyses were performed three times, and then the arithmetic mean was calculated from the obtained concentrations. Based on the results obtained, the concentration of mono–15 and 15–4–15 additives were selected for further tests with lubricants. The results are shown in Figure 9 and Figure 10.

##### The Preparation of Analytical Samples for the Evaluation of Lubricants Containing Surface-Active Additives

A potassium diphosphate buffer (KH_2_PO_4_ p.a., Chempur) in the ratio of 1:800 (v) with 0.25M KH_2_PO_4_ stock solution (pH = 7.0) was used in the tests and sterilised. In the next step, a bacterial inoculum was prepared at a concentration of (1.5–3.0) · 10^8^ CFU/cm^3^ (CFU, colony forming unit) by inoculating the sterile phosphate buffer with a suspension of overnight cultures of *S. aureus* and *S. enteritica*. The prepared suspension of bacteria with this concentration was used as a working solution. The greases, weighing 1.0 ± 0.1 g and containing the appropriate weight percentage of the tested additives, were transferred to sterile Erlenmeyer flasks with a capacity of 250 cm^3^. Then, 50 cm^3^ of the working solution with the tested bacteria was transferred to each flask and shaken at room temperature at 180 rpm for 1 h. After this time, the resulting suspension was inoculated via serial dilution on the plates with tryptic soy agar growth medium (TSA, VWR). The plates were incubated at 37 °C for 24 h in order to determine the CFU value. A solution of the working suspension of bacteria (as the negative control sample) and the grease without additives (as the positive control sample) were used as the control samples. A positive test was a grease without an additive, the use of which allowed the assessment of how the addition of mono–15 and 15–4–15 affected the antibacterial properties. The analyses were performed three times, and the CFU/cm^3^ results were averaged and the percentage reduction in the viability of the analysed bacterial strains compared to the negative control was calculated according to Formula (1).

#### 4.1.4. Tribological Tests

The basic tribological experiment was carried out using a T-02 four-ball apparatus produced by the Łukasiewicz Research Network—Institute for Sustainable Technologies in Radom in accordance with the standardised requirements. Tribological tests were carried out according to the WTWT-94/MPS-025 Military Temporary Technical Requirements standard for testing the anti-wear properties of propellant and lubricant materials. The test makes it possible to determine the limiting load of wear for the tested sample (392.4 N) and the diameter of the wear mark on the stationary balls. The test was carried out at a temperature of 20 °C, with load of 392.4 N for 3600 s and a spindle speed of 500 rpm. The Goz/40 parameter was calculated according to the following formula:

G_oz_/40 = 0.52 × 392.4/d^2^

where 392.4 is the load of the friction node in [N], and d is the average diameter of the flaw formed on the steel balls used for the examination.

For each sample, tribological tests on the T-02 four-ball apparatus were carried out in three repetitions.

#### 4.1.5. Oxidative Stability

Oxidative stability was determined in accelerated oxidation tests using the PetroOxy^TM^ apparatus by Petrotest Instrument. The tested lubricants were oxidised with a stream of oxygen at a temperature of 80 °C, while maintaining the same sample weight of 10 ± 0.1 g. The following conditions were applied: a filling pressure of 700 kPa and an oxygen pressure of 8 bar (800 kPa). The time needed to obtain the maximum pressure drop in the measuring chamber by 10% was determined.

#### 4.1.6. Corrosion Test

Corrosion tests were performed on copper plates according to PN-EN ISO 2160:2004. According to the standard, the tests lasted 3 h. The tests of oils were carried out at a temperature of 50 °C, and the tests of lubricants were conducted at a temperature of 100 °C. The assessment of the corrosion state of the copper plate after the tests consisted of comparing the appearance of the copper plate with the corrosion pattern.

### 4.2. Synthesis Procedures

#### 4.2.1. Synthesis of [3-(*N*,*N*-dimethylamine)propyl]hexadecanamide

The synthesis conditions were based on data from the literature [70]. In a three-neck flask equipped with a condenser, a dropping funnel, a thermometer, and a dipole stirrer, 0.5 mol of 3-(*N*,*N*-dimethylamine)propylamine (Merck, for synthesis) and 700 mL dichloromethane (Avantor, pure p.a.) were rapidly mixed under nitrogen at an ambient temperature. Next, 0.5 mol of palmitoyl chloride (Merck, for synthesis) was added dropwise. After the addition of palmitoyl chloride, the reaction mixture was stirred for 30 min and left under nitrogen until the next day. Next, dichloromethane was evaporated under a vacuum. The reaction mixture was treated with 600 mL 1M NaOH and stirred vigorously; then, it was extracted using a chloroform/methanol mixture (1:2 *v*/*v*). All solvents from the organic phase were evaporated and, afterwards, the product was dried over dehydrated magnesium sulphate. The raw product was dissolved in ethyl acetate and crystallised at 0 °C. A white, solid product was obtained with a 74.5% yield.

#### 4.2.2. Synthesis of 15–4–15 Gemini QAC

In a three-necked flask equipped with a condenser, a dropping funnel, a thermometer, and a dipole stirrer, 0.24 mol of the previously obtained [3-(*N*,*N*-dimethylamine)propyl]hexadecanamide and 450 mL acetonitrile (BDH, 98%) were rapidly stirred under nitrogen at 82 °C. Next, 0.11 mol of 1,4-dichlorobutane (Acros, 99%) was added dropwise and mixed for 18 h at 82 °C. Then, acetonitrile and non-reacted 1,4-dichlorobutane were evaporated under a vacuum. The raw product was dissolved in an acetonitrile/toluene mixture (1:1 *v*/*v*) and crystallised. A pale yellow solid product was obtained with a 29% yield.

#### 4.2.3. Synthesis of mono–15 QAC

The synthesis conditions were based on data from the literature [71]. In a three-necked flask equipped with a condenser, a dropping funnel, a thermometer, and a dipole stirrer, 0.24 mol of the previously obtained [3-(*N*,*N*-dimethylamine)propyl]hexadecanamide and 450 mL acetonitrile (BDH, 98%) were rapidly mixed under nitrogen at 82 °C. Next, 0.27 mol of 1-chlorobutane (Sigma Aldrich, 99%) was added dropwise and mixed for 18 h at 82 °C. Then, acetonitrile and non-reacted 1-chlorobutane were evaporated under a vacuum. The raw product was dissolved in an acetonitrile/toluene mixture (1:1 *v*/*v*) and crystallied. A pale yellow solid product was obtained with a 46% yield.

## 5. Conclusions

The introduction of additives (mono–15, 15–4–15) to vegetable oil and grease is highly effective in slowing down the oxidation process. Vegetable oil stabilised with these additives is characterised by much higher oxidative stability compared to unstabilised oil.

The obtained results indicate that the compounds produced have a bactericidal effect, and this effect is better for the grease with the addition of 15–4–5 against both types of bacteria (Gram-positive and Gram-negative).

In summary, the tribological tests carried out under boundary friction conditions showed a relationship between the composition of the lubricants and the lubricating properties characterised by the following parameters: limit wear load G_oz_ and the average diameter of the wear-traced oxidation process. The obtained results confirmed not only the bactericidal effect of the developed additives, but also their beneficial effect as anti-wear additives and oxidation inhibitors. In conclusion, it was proven that the analysed additives had antibacterial properties, and tribological tests confirmed that the scar diameter of the evaluated lubricating compositions indicates a low value of wear, which corresponds to the limiting load of wear value G_oz_/40.

## Figures and Tables

**Table 1:** Gemini QACs and their antimicrobial activity, expressed as MIC (µg/mL) [46].

	Gemini QACs					
	12–2–12	12–3–12	12–4–12	14–2–14	14–3–14	14–4–14
*S. aureus*	6	6	1.5	8	6	20
*P. aeruginosa*	200	200	200	>800	>800	>800
*E. coli*	50	50	50	500	600	600

**Table 2:** Gemini QACs and their antimicrobial activity, expressed as MIC (mmol/L) [47].

		Gemini QACs				
		16–2–16	16–4–16	16–6–16	12–2–12	12–4–12
Gram (+)	*B. subtilis*	0.13	0.14	0.19	0.40	0.39
	*S. aureus*	0.20	0.19	0.32	0.33	0.31
Gram (−)	*K. pneumonia*	0.20	0.19	0.32	0.40	0.39
	*S. paratyphi type B*	0.28	0.53	0.51	0.33	0.31
	*A. niger*	0.10	0.10	0.19	0.24	0.16

**Table 3:** Gemini QACs and their antimicrobial activity, expressed as the inhibition zone (mm) [48].

	Mono and Gemini QACs					
	14–Mono	14–3–14	14–6–14	18-Mono	18–3–18	18–6–18
*P. aeruginosa*	-	19	13	9	12	9
*B. subtilis*	15	12	15	-	-	11
*S. aureus*	16	15	16	11	10	10

**Table 4:** Molecular peaks obtained from the mass spectrometry of the [3-(*N*,*N*-dimethylamine)propyl]hexadecanamide sample.

Product (GC)	Molecular Weight, Da	Structure	Name	Content, %
1	340	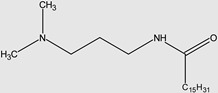	[3-(*N*,*N*-dimethylamine)propyl]hexadecanamide	97.6
2	338	N/A	N/A, probably unsaturated product 1	2.4

**Table 5:** Molecular peaks obtained from mass spectrometry of the 15–4–15 sample.

Product (GC)	Molecular Weight, Da	Structure	Name
1	100	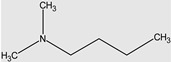	*N*,*N*-dimethylbutylamine
2	296.5	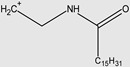	N/A
3	341.4	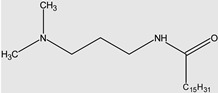	[3-(*N*,*N*-dimethylamine)propyl]hexadecanamide
4	431.0	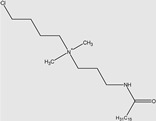	N/A
5	771.7	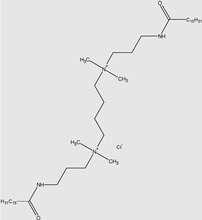	15–4–15 (single Cl^−^)

**Table 6:** Molecular peaks obtained from the mass spectrometry of the 15–4–15 sample.

Product (GC)	Molecular Weight, Da	Structure	Name
1	296.5	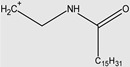	N/A
2	341.4	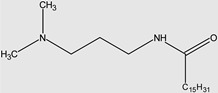	[3-(*N*,*N*-dimethylamine)propyl]hexadecanamide
3	397.4	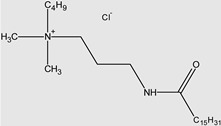	mono–15 QAC (without Cl^−^)

**Table 7:** Lubricating properties, anticorrosive properties, and oxidation stability of lubricants.

Lubricant	G_oz_/40, N/mm^2^ (WTWT-94/MPS-025)	Wear Diameter, d, mm (WTWT-94/MPS-025)	Oxidation Induction Time, at 80 °C, OIT, h (ASTM D 942-02)	Corrosion Degree (PN-EN ISO 2160:2004)
Oil A	383 ± 27	0.73 ± 0.04	1.2	1 a
Oil A + mono–15	455 ± 32	0.67 ± 0.05	7.2	1 a
Oil A + 15–4–15	531 ± 42	0.62 ± 0.04	8.1	1 a
Grease A	886 ± 51	0.48 ± 0.01	36.7	1 a
Grease A + mono–15	1008 ± 128	0.45 ± 0.02	40.3	1 b
Grease A + 15–4–15	1157 ± 147	0.42 ± 0.02	41.9	1 b

## Data Availability

The data presented in this study are available upon request from the corresponding author.
